# Surgical sterilization of free-ranging capybaras (*Hydrochoerus hydrochaeris*): “Passos Nunes” uterine horn ligature

**DOI:** 10.1590/1984-3143-AR2022-0029

**Published:** 2022-08-08

**Authors:** Fernanda Battistella Passos-Nunes, Fabiana Morse Gosson Jorge, Mariana Passos Nunes, Alexandre Zanetti Nunes, Pedro Nacib Jorge-Neto, Antonio Chaves de Assis, Marcelo Bahia Labruna, Cristiane Schilbach Pizzutto

**Affiliations:** 1 Departamento de Reprodução Animal, Faculdade de Medicina Veterinária e Zootecnia, Universidade de São Paulo, São Paulo, SP, Brasil; 2 AZ Nunes & Cia, Itu, SP, Brasil; 3 Instituto Reprocon, Campo Grande, MS, Brasil; 4 Departamento de Cirurgia, Faculdade de Medicina Veterinária e Zootecnia, Universidade de São Paulo, São Paulo, SP, Brasil; 5 Departamento de Medicina Veterinária Preventiva e Saúde Animal, Faculdade de Medicina Veterinária e Zootecnia, Universidade de São Paulo, São Paulo, SP, Brasil

**Keywords:** spotted fever, surgical sterilization, reproductive management

## Abstract

Capybaras are the primary hosts of *Amblyomma sculptum* tick, vectors of *Rickettsia rickettsia* bacteria, and the zoonotic agent of Brazilian Spotted Fever (BSF). In this context, contraceptive methods have been suggested for population control in order to reduce the number of free-ranging capybaras cohabiting with humans in urban and rural areas and acting as disease amplifiers. To maintain the group's expected behavior and social hierarchy, sterilization techniques that preserve the gonads are recommended. On 126 female capybaras in the Brazilian state of São Paulo, a new surgical technique named “Passos Nunes” uterine horn ligature was performed after adequate general anesthesia. It achieved effective surgical sterilization, with an incision length of about 3 cm in the periumbilical linea alba, cranial to the pubis. After entering the abdominal cavity, the urinary bladder is pulled laterally to access the uterine horns and the cervix. The uterine horn is folded up, forming a strap; the distal portion of the strap is ligated and its distal end sectioned. The exact process is performed on the opposite horn. After the surgical procedure, the musculature is sutured in a sultan pattern and the subcutaneous tissue with a horizontal mattress pattern. The skin is sutured in a separate simple format, using nylon 2.0 for all steps. The wide exposure of the uterine horns facilitates the confirmation of pregnancy, allowing the surgeon to choose between salpingo hysterectomy or ligature of the uterine horns. The present study presents a new technique of surgical sterilization that can be used in female free-ranging mammals in which maintenance of the gonads is recommended, and births of offspring should not occur.

## Introduction

On the entire Brazilian territory, capybaras (*Hydrochoerus hydrochaeris*) cohabit with humans in both urban and rural areas, causing health authorities to worry about the transmission of Brazilian Spotted Fever (BSF). Capybaras are the primary hosts of *Amblyomma sculptum* ticks, which are the vectors of *Rickettsia rickettsii* bacteria, and BSF agents (Passos-Nunes et al., 2020). Environmental authorities have been proposed population control techniques (São Paulo, 2016) to reduce tick carriage (Polo et al., 2017) because this is a highly lethal disease if not treated promptly (Araújo et al., 2015).

In consideration of the gregarious nature of these animals as a result of their hormone-dependent hierarchy (MacDonald & Herrera, 2013), the present study proposes a new surgical method of sterilization that preserves the gonads. The procedure involves ligature of uterine horns, transfixion and partial incision of the distal end of the horn. It allows population control with minimal incision (less than 50mm), reduced surgical time when compared to the traditional tubal ligation methods, as described by Pradere et al. (2006) and Yanai et al. (2022), a good exposure of uterine horns, facilitating hysterectomy or hysterotomy when pregnancy is discovered, and efficient sterilization in field conditions, making it an alternative surgical sterilization technique to partial salpingectomy.

## Methods

This experiment was conducted with 126 free-ranging adult female capybaras in population management programs carried out in eight different municipalities (Table 1) in the State of São Paulo (Brazil), previously authorized by the Department of Fauna of the Secretariat of Infrastructure and Environment of the State of São Paulo (DeFau/SMA/SP) (Authorizations: Itu nº 0000033882/19; Cajamar nº 0000078525/17; Porto Feliz nº 0000080229/17; Vineyard nº 0000008605/21; Louveira nº 3700958/20; Atibaia nº 0000014629/ 21; Salto nº 0000062884/20; and Tatuí nº 000001803/18). The study was also authorized for scientific activities by SISBIO/ICMBio/MMA (nº 79881) and approved by CEUA/FMVZ/USP (no. 1106020919).

**Table 1:** Number of females submitted to partial cornotomy of “Passos-Nunes” compared to the total number of animals counted in eleven enterprises located in eight different municipalities, São Paulo, 2022.

**Places**	**Number of enterprises**	**Number of females who underwent partial cornotomy of “Passos-Nunes”**	**Number of animals in the group**
**Tatuí**	**1**	**19**	**71**
**Itu**	**3**	**32**	**73**
**Porto Feliz**	**1**	**15**	**73**
**Cajamar**	**1**	**13**	**34**
**Salto**	**1**	**5**	**21**
**Vinhedo**	**1**	**9**	**19**
**Louveira**	**1**	**14**	**23**
**Atibaia**	**1**	**19**	**35**

Female capybaras were captured with bait, as described in the scientific works (Nunes et al., 2019; Passos-Nunes et al., 2020). The surgical procedures were performed in accordance with the work plans authorized by DeFau/SMA/SP by two surgeons and auxiliary veterinarians working in cold-floor rooms with adequate lighting and ventilation for field procedures. Total surgical time (skin incision to completion of skin closure), incision length following suture, and complications were recorded.

Chemical restraint was performed using anesthetic darts (Dist inject system) fired with a blowpipe containing ketamine hydrochloride (5 to 10 mg/kg; intramuscularly (IM); Ketalex, Venco, PR, Brazil) and xylazine hydrochloride (0.5 to 1.0 mg /kg; IM; Sedalex, Venco, PR, Brazil). Immediately after chemical restraint, morphine sulfate (2 to 10 mg/kg; IM; 70 Hipolabor, MG, Brazil) was administered for analgesia

Within five to ten minutes of the anesthetic dart being administered, the animals begin to exhibit sedation signs such as chewing and staggering gait. During all surgical periods, heart rate, respiratory rate, pulse oximetry, and body temperature were measured using a portable multiparameter device (AM 6 100, Vet Care) to monitor physiological monitoring parameters. Moreover, pupillary and corneal reflexes were monitored every five minutes.

For the trichotomy, the females were positioned in dorsal recumbency. The surgical site was cleaned with 2% chlorhexidine (Riohex, Rioqumica) and 5% chlorhexidine (Riohex, Rioqumica) for antisepsis. On the incision line, 2% lidocaine hydrochloride (0.1 mg/kg; intradermally (ID); Xylestesin, Cristália, SP, Brazil) was used to administer local anesthesia. Before the surgical procedure, the females were treated with antibiotics (0.1 mL/kg; im; Agrovet 5,000,000 with procaine benzylpenicillin 3,750,000 I.U.; potassium benzylpenicillin 1,250,000 I.U.; streptomycin sulfate 2.0 g per 15 mL; Elanco), antiinflammatory (Meloxicam, 0.6 mg/kg; sc; Maxican 2%, Ouro Fino, Brazil) and antiparasitic (Ivermectin 1%, 0.02 mL/kg Merial, Brazil).

On the linea alba, a 3-centimeter incision was made in the skin immediately above the pubis. The *panniculus carnosus* muscle was dissected, and the *linea alba* and *rectus abdominis* muscles were incised, along with its profound sheet, to expose the abdominal cavity. The urinary bladder was subsequently pulled laterally to expose the left and the right uterine horn.

**Figure 1:**
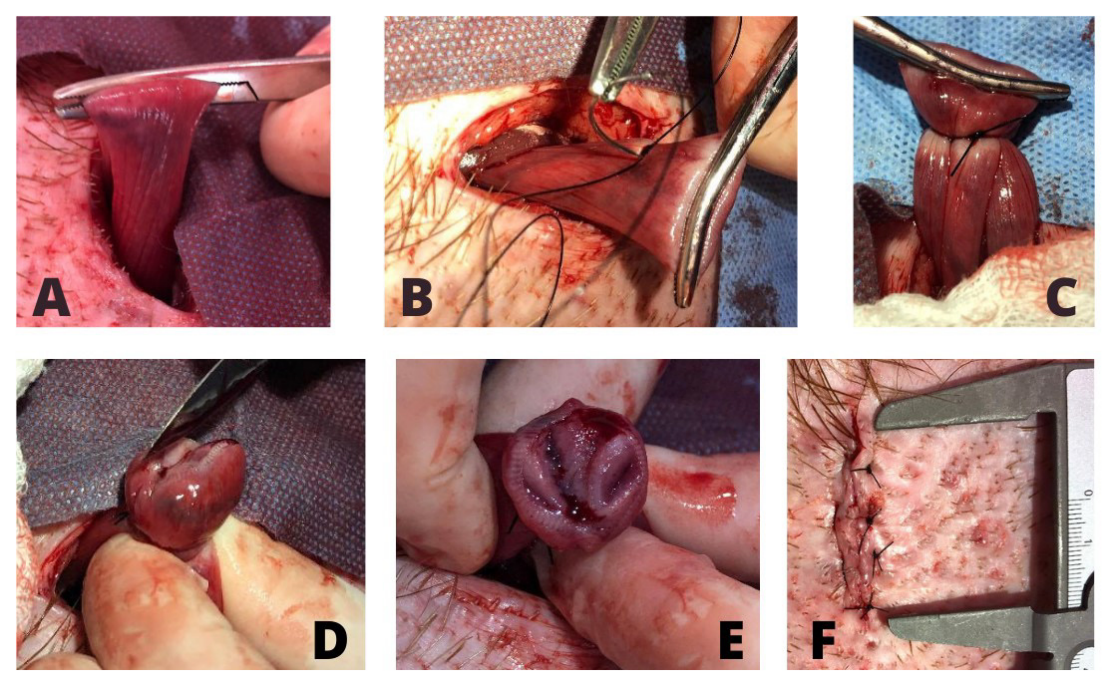
(A) exposure of the uterine horn; (B) transfixion using nylon mesh; (C) transfixion concluded with both sides of the horn ligated; (D) incision of the distal part of the ligated horn; (E) final aspect of the ligated horn after incision; (F) final aspect of the surgical incision after skin suture.

The proximal third of the uterine horn is clamped with Crile hemostatic forceps. The transfixion and ligature were carried out in the form of an “8” using nylon 2.0 suture thread (Atramat), joining the ends immediately cranial and caudal to the forceps to form a loop. Following ligation, a partial section of the distal end of the loop, where the hemostat was located, was performed (Figure 1D). The same procedure is performed on the opposite horn of the uterus.

The aponeurosis of the abdominal muscles and the panniculus carnosus muscle were sutured separately using a sultan pattern. The subcutaneous tissue was sutured using a horizontal mattress pattern, and the skin was sutured using a simple pattern, all with nylon 2.0. Over the skin suture, surgical glue (Tissue Aid TM, 2-Octyl Cyanoacrylate) was applied (Figure 1F).

The return of the anesthetic occurs between 10 and 15 minutes following a routine surgical procedure. After complete anesthetic recovery (normal gait, normal protective reflexes, and response to outside stimuli), the animals were returned to the fenced enclosure where they were initially baited and captured before being released. Maintaining these animals in captivity increases the risk of stress-induced tympany and the likelihood of intragroup conflict (Rosenfield et al., 2019b).

## Results

One hundred twenty-six (126) female capybaras were sterilized using the “Passos-Nunes” horn ligature technique between 2018 and 2020. Only pregnant mares requiring a hysterectomy or hysterotomy were observed to experience surgical complications. In these cases, there was an abundance of hemorrhage due to the rich vascularity of the mesometrium, but no blood transfusion was necessary. In routine surgeries, the surgical time varied between 20 and 25 minutes, and the recovery time ranged between 10 and 15 minutes.

These groups were observed and followed for a variable period of time (two to five years), and in the last three years, no newborns of the sterilized females were observed. All of the animals were a part of population control programs, and their random recapture hindered the collection of data. All recaptured capybaras exhibited healthy wound healing at the incision site.

## Discussion

Brazilian Spotted Fever is an issue of public health. Therefore, surgical sterilization is recommended as a method of population control for free-living capybaras (São Paulo, 2016), with the goal of stabilizing the population without disrupting family hierarchies (Rodrigues et al., 2017).

Capybaras must have their gonads preserved in order to maintain cohesive groups and to facilitate post-surgical follow-up (Passos-Nunes et al., 2020; Silva et al., 2018). Population control by *in situ* sterilization of capybaras reduces the methods of removal of this species by euthanasia in endemic areas of BSF, bringing a synergy between different lines of research in accordance with the modern concept of One Health (Pizzutto et al., 2021). Additionally, reproductive management and the reduction of euthanasia for population control prevent social unrest and ecological imbalance.

Immunocontraceptive vaccines have been proposed as an effective contraceptive strategy for male capybaras, preserving the alpha male's social behavior (Rosenfield & Pizzutto, 2019), with the benefit of being less invasive (Rosenfield et al., 2019a) and also being a strategy for BSF control (Rosenfield et al., 2019c).

Among the surgical sterilization techniques described for capybaras, tubal ligation and vasectomy are viable options that allow the group structure and social behavior to be preserved (Passos-Nunes et al., 2020; Rodrigues et al., 2017). In addition, it decreased the amplification of *R. rickettsii* bacteria in BMB-endemic areas, thereby contributing to public health (Passos-Nunes et al., 2020). In contrast to the “Passos Nunes” technique, however, it does not permit extensive exposure of both uterine horns, necessitating hysterectomy or hysterotomy in pregnant mares, a crucial issue in endemic areas or with risk of BSF transmission, where the birth of seronegative pups maintains the BSF cycle. During bacteremia, newborns are susceptible to transmitting *R. rickettsii* to approximately 25% of ticks that parasitize capybaras, according to Souza et al., (2009).

This method allowed for a brief surgical and anesthetic period. In addition, a small incision of about three centimeters contributed to a faster recovery and good exposure of both uterine horns, allowing the surgeon to choose between performing a cesarean section or hysterectomy on pregnant capybaras.

## Conclusion

The “Passos-Nunes” horn ligature described as a new technique for reproductive control of capybaras proved effective for *in situ* free-ranging capybaras population control. It offers advantages over tubal ligation techniques, such as broad exposure of uterine horns, effortless performance of hysterectomy or hysterotomy when pregnancy is detected, and short total surgical time due to the fact that it is performed through a single surgical incision.
